# Impact of the COVID-19 Pandemic on Children with ASD and ADHD in Northern Greece: A Pilot Study

**DOI:** 10.3390/brainsci15111212

**Published:** 2025-11-10

**Authors:** Efterpi Pavlidou, Anna Samara, Sofia Michailidou, Maria Kinali, Martha Spilioti, Nafsika Ziavra

**Affiliations:** 1 Speech and Language Therapy Department, School of Health Sciences, University of Ioannina, 45000 Ioannina, Greece; annasa.25@gmail.com (A.S.); sofiamich01@yahoo.gr (S.M.); nziavra@uoi.gr (N.Z.); 2 Department of Brain Sciences, Imperial College London, London SW7 2AZ, UK; m.kinali@imperial.ac.uk; 3 Neurology Department, AHEPA Hospital, Aristotle University of Thessaloniki, 54636 Thessaloniki, Greece; marthags@auth.gr

**Keywords:** COVID-19, pandemic crisis, ADHD, ASD, children

## Abstract

Background/Objectives: The COVID-19 pandemic profoundly disrupted the daily lives of children with neurodevelopmental disorders, particularly Autism Spectrum Disorder (ASD) and Attention-Deficit/Hyperactivity Disorder (ADHD). Lockdowns, therapy interruptions, and reduced access to educational and healthcare services significantly affected developmental progress and family functioning. This pilot study aimed to assess the long-term impact of the pandemic on children with ASD and ADHD in Northern Greece and to explore consequences for their families in the post-pandemic period. Methods: Parents or legal guardians of 72 children (ages 2–17 years) with confirmed diagnoses of ASD (*n* = 57) or ADHD (*n* = 15) participated. A structured 25-item questionnaire captured information on developmental, psychological, and social functioning, family well-being, therapy disruption, screen use, and access to online support. Data were collected across urban, semi-urban, and rural areas of Northern Greece over six months. Descriptive and inferential analyses were performed. Results: Most participants were boys (77.8%) and in primary school (73.6%). Common co-occurring conditions included learning difficulties (33.3%), anxiety (8.3%), and epilepsy (6.9%). Nearly half of families (45.8%) reported therapy reductions exceeding 70%, while 29.2% accessed online therapy, often with limited perceived effectiveness. New behavioral symptoms emerged in 45.8% of children, including irritability, anxiety, and emotional instability. Parental psychological distress was reported by 29.2% of caregivers. Screen time increased in over 90% of cases, and 87.5% of parents perceived the pandemic as negatively affecting their child. Financial strain was noted by 37.5% of families. Conclusions: The findings highlight the significant developmental, psychological, and social consequences of the COVID-19 pandemic for children with ASD and ADHD and their families. Service disruptions, unmet therapeutic needs, and increased caregiver burden emphasize the urgency of sustainable, flexible care models. Strengthening telehealth, integrating community-based interventions, and enhancing educational accommodations are essential for resilience in the post-pandemic era.

## 1. Introduction

The COVID-19 pandemic, caused by the SARS-CoV-2 virus, has had profound and widespread consequences on global health systems, particularly affecting vulnerable populations such as children with neurodevelopmental disorders, including Autism Spectrum Disorder (ASD) and Attention-Deficit/Hyperactivity Disorder (ADHD) [1,2]. The implementation of restrictive public health measures—including school closures, limitations on travel and/or stay-at-home orders, and reduced access to healthcare—interfered with vital routines that foster cognitive, social, and emotional growth, and hindered access to therapeutic interventions crucial for these children [2,3].

Increasing evidence points to a marked worsening of behavioral, emotional, and psychosocial outcomes in children with ASD, particularly boys, including greater screen exposure, intensification of stereotyped behaviors, emotional regression, and motor tics [4,5,6]. In the same context, children with ADHD experienced heightened levels of anxiety, irritability, inattention, and sleep disturbances, all of which were amplified by social isolation and the suspension of structured educational and therapeutic services [7,8].

Multiple studies have highlighted that pandemic-related containment strategies adversely affected not only children with special needs but also their families [9,10]. The abrupt discontinuation of essential interventions, coupled with elevated parental stress and limited professional support, imposed significant caregiving burdens [11]. Children with co-morbidities or sensory integration difficulties faced additional challenges in adhering to preventive health measures, thereby heightening their vulnerability [4,12]. Importantly, parental anxiety was found to be closely associated with behavioral regression in children [13]. While tele-health and pharmacological support were adopted as interim solutions, they often fell short as substitutes for in-person care, emphasizing the need for flexible, child-specific support systems [8,14]. The cumulative psychological burden stemming from prolonged lockdowns, economic hardship, and disrupted routines calls for renewed policies focused on integrated and holistic care models [15]. Evidence-based recommendations highlight the importance of sustaining therapeutic continuity and empowering caregivers through targeted education; in tandem, scaling up tele-psychiatry services is essential to mitigate long-term negative outcomes [16]. Importantly, routine disruption was identified as a key contributor to emotional dysregulation in both ASD and ADHD populations [15]. Therefore, health systems must respond to the multifactorial impact of the pandemic by investing in targeted interventions and building resilient care infrastructures for neurodiverse children and their families [7,13].

### Aim of the Study

This pilot quantitative study aims to assess the impact and long-term impact of the COVID-19 pandemic on children diagnosed with ASD and ADHD during the post-pandemic period. The specific objectives of the study are to:

(a) evaluate the magnitude and nature of the pandemic’s effects on developmental functioning, psychosocial adjustment, and adaptive outcomes in children with ASD and ADHD, acknowledging the cross-sectional design;
(b) examine the impact of the pandemic on the quality of life and psychosocial well-being of their families; and
(c) investigate the long-term clinical and adaptive outcomes for these children within the evolving landscape of post-pandemic healthcare and social environments.

The post-pandemic period is defined as the period when disease activity has diminished and returned to levels typical of viral infection.

## 2. Patients and Methods

### 2.1. Sample

The study population consisted of parents of children with formally diagnosed neurodevelopmental disorders. Eligibility criteria required that participants be the parents or legal guardians of children aged 2 to 17 years who had received a confirmed clinical diagnosis of a neurodevelopmental disorder. Written informed consent was obtained from all participants prior to their inclusion in the study. To safeguard anonymity and ensure confidentiality, all questionnaires were completed anonymously (Supplementary Material are available).

### 2.2. Ethical Approval

The study was conducted in accordance with the Declaration of Helsinki. Ethical approval was granted by the Research Ethics Committee of the University of Ioannina. Written informed consent was obtained from all participants prior to inclusion. The present study was conducted as part of an undergraduate research thesis in the Department of Speech and Language Therapy at the University of Ioannina.

### 2.3. Data Collection Procedure

The research followed an approved protocol and was implemented in two sequential phases. During the first phase, formal approval was secured from the relevant authorities. In the second phase, a structured questionnaire was administered in person to each participant—either the parent or the primary caregiver—under the guidance of trained research personnel.

Data collection and processing were conducted over a six-month period. Completion of each questionnaire required approximately 15 min. Upon collection, participant data were screened according to exclusion criteria, which encompassed children under two years of age and those with typical (non-pathological) neurodevelopmental profiles. Subsequently, all responses were anonymized, coded, entered into a secure database, and prepared for statistical analysis.

### 2.4. Study Setting and Duration

The study was conducted over a six-month period between December 2022 and June 2023 across various urban and semi-urban locations in Northern Greece. Participants were recruited from therapeutic centers and Creative Activity Centers for Children (CACC) during routine therapy visits with their children.

### 2.5. Research Instruments

We employed a structured 25-item questionnaire to capture post-pandemic impacts in key domains for children with ASD/ADHD and their families. Items covered: (i) therapy access/disruption and service use, (ii) behavioral and emotional symptoms, (iii) social communication/interaction and adaptive functioning, (iv) sleep and media/screen use, (v) educational participation/remote support, and (vi) caregiver well-being and family routine. Response formats included Likert-type scales (e.g., 5-point agreement or frequency), multiple-choice categories, and yes/no items; coding keys are provided in Supplementary File S1. The instrument underwent expert review for face/content validity prior to use; minor wording refinements followed pilot administration to ensure clarity. Where applicable, internal consistency (Cronbach’s alpha) was evaluated for multi-item domains and is reported in the Supplement. Administration took approximately 15 min by trained research personnel. The questionnaire was developed by the Department of Speech and Language Therapy at the University of Ioannina and received approval from the Research Ethics Committee of the University of Ioannina.

Given the cross-sectional design, results reflect associations at a single post-pandemic timepoint and do not infer within-child change over time. Based on the study experience, future versions of the questionnaire will include more detailed instructions to facilitate uniform interpretation by respondents.

### 2.6. Statistical Analysis

Data analysis was performed using IBM SPSS Statistics (Version 28.0.1.0). Analyses were primarily descriptive, consistent with the pilot design. Categorical variables are presented as counts and percentages; continuous variables as means ± SD or medians with interquartile ranges, as appropriate. Where relevant, 95% confidence intervals are reported. No confirmatory hypothesis testing was pre-specified. Any exploratory comparisons, when presented, are explicitly labeled and used only to inform future hypothesis generation (e.g., Fisher’s exact test for categorical variables with small cell counts; Wilcoxon rank-sum test for non-normal continuous variables). All analyses were conducted using standard statistical software.

### 2.7. Psychometric Considerations

Internal consistency (Cronbach’s α) was calculated for multi-item domains. Face/content validity were established via expert review and pilot testing for clarity. Given the pilot scope and sample size, we did not undertake full construct validation (EFA/CFA), which we plan for a larger follow-up study with independent samples. Accordingly, we interpret composite scores cautiously and emphasize estimation over inference.

## 3. Results

A total of 72 children participated in the study, of whom 56 were boys (77.8%) and 16 were girls (22.2%), reflecting the known gender disparity in the prevalence of neurodevelopmental disorders such as ASD and ADHD.

### 3.1. Age Distribution

The sample included 17 children (23.6%) aged 2–5 years, 36 (50%) aged 5–9 years, 9 (12.5%) aged 9–13 years, and 10 children (13.9%) aged 13–17 years, with the 5–9 age group constituting the largest proportion. Table 1 displays demographic characteristics.

### 3.2. Educational Status

At the time of data collection, 53 children (73.6%) were enrolled in primary education, 10 (13.9%) in secondary education, while 9 children (12.5%) were not attending any formal schooling.

### 3.3. Residential Location

With respect to residence, 29 respondents (40.3%) lived in large urban areas, 28 (38.9%) in small towns, and 15 (20.8%) in rural settings.

### 3.4. Parental Educational Background

Most parents (n = 57; 79.2%) had attained tertiary education, 12 (16.7%) had completed secondary education, and 3 parents (4.2%) had received only primary education.

### 3.5. Diagnostic Profiles

Of the children studied, 57 (79.2%) had a diagnosis of ASD, while 15 (20.8%) had ADHD. Table 2 shows the diagnosis and co-morbidity profile.

### 3.6. Co-Morbidities

Among the sample, 24 children (33.3%) were reported to have learning difficulties, 6 (8.3%) had an anxiety disorder, and 5 (6.9%) were diagnosed with epilepsy. Multiple co-morbid conditions were involved in 8 cases (11.1%), while 29 parents (40.3%) indicated the absence of any additional diagnoses.

### 3.7. Child and Parent Psychological Status During the Pandemic

In terms of perceived psychological well-being of the children during the pandemic, 11 parents (15.3%) rated their child’s condition as very good, 50 (69.4%) as satisfactory, and 11 (15.3%) as poor. Regarding their own psychological state, 9 parents (12.5%) reported feeling very good, 42 (58.3%) satisfactory, and 21 (29.2%) described significant psychological distress.

### 3.8. Use of Mental Health Services

A total of 29 parents (40.3%) reported that they felt the need to consult a psychologist during the pandemic, while the remaining 43 parents (59.7%) did not perceive such a need. Telehealth uptake remained limited relative to in-person services, and most caregivers perceived online sessions as less effective than conventional therapy. Reported therapy reductions were substantial during the peak pandemic period, with partial recovery thereafter. Caregiver psychological strain was commonly endorsed. The distribution of comorbidities indicated that approximately 60% of children had at least one co-occurring condition, consistent with prior literature. Exact values are provided in Table 1.

### 3.9. Therapy Disruption

Substantial reductions in therapeutic services were observed: 33 parents (45.8%) reported therapy reductions exceeding 70%, 16 (22.2%) indicated a 50% reduction, and 23 (31.9%) reported reductions of less than 30%.

### 3.10. Emergence of Behavioral Symptoms

New behavioral issues were reported by 33 parents (45.8%) during the pandemic. Among these, 13 (18.1%) observed irritability or aggression, 10 (13.9%) reported anxiety, 5 (6.9%) noted emotional instability, and 5 (6.9%) identified co-occurring symptoms. The remaining 39 parents (54.2%) indicated that no new behavioral symptoms emerged during this period.

### 3.11. Impact on Family Routine

Most respondents (n = 59; 81.9%) stated that the pandemic significantly disrupted daily routines. Ten parents (13.9%) were unsure of the impact, while only three (4.2%) reported no change in routine.

### 3.12. Access to and Perceptions of Online Therapy

Only 21 parents (29.2%) reported that their child had access to online therapy, while the majority (70.8%) did not engage in virtual sessions. Among the subset who did, 13 parents (18.1%) rated the sessions as less than 50% effective, 4 (5.6%) considered them fully effective, and another 4 (5.6%) found them entirely ineffective. The remaining participants did not respond to this question, most likely due to non-utilization.

### 3.13. Digital Media Use

An increase in screen time was observed in the vast majority of cases: 34 parents (47.2%) reported a marked increase in the use of digital devices, 32 (44.4%) noted a moderate increase, and only 6 (8.3%) saw no substantial change.

### 3.14. Academic Support

Sixteen parents (22.2%) indicated that they arranged additional private lessons to support their child’s academic progress during the pandemic, whereas the majority (n = 56; 77.8%) did not pursue supplemental instruction.

### 3.15. Parent–Child Interaction

Nearly half of the respondents (48.6%) reported spending quality and productive time with their child during lockdown. Another 44.4% indicated that the time spent was moderately productive but less than they would have liked, while 6.9% stated that they were unable to engage in meaningful or productive interactions with their child.

### 3.16. Parental Perceptions of Pandemic Impact

When asked whether the pandemic had a negative impact on their child, 63 parents (87.5%) responded affirmatively, while 9 parents (12.5%) stated that their child had not been affected. Notably, no parent reported a positive impact.

### 3.17. Sleep and Neurological Symptoms

Sleep disturbances were reported in 17 children (23.6%), while 55 (76.4%) showed no such difficulties. Regarding neurological or speech-related symptoms that emerged for the first time during the pandemic, 10 parents (13.9%) reported speech regression, 7 (9.7%) noted the onset of tics, and 4 (5.6%) observed new stuttering. The majority (n = 51; 70.8%) did not report any of these symptoms.

### 3.18. Consultation with Mental Health Professionals

A total of 13 parents (18.1%) consulted a psychologist or child psychiatrist at least once during the pandemic. An additional 6 parents (8.3%) reported regular ongoing consultations, while the majority (n = 53; 73.6%) did not seek professional mental health support for their child.

### 3.19. Economic Impact

Regarding financial status during the pandemic, 44 parents (61.1%) reported stability, 27 (37.5%) experienced a decline in their financial condition, and only one parent (1.4%) reported an improvement. Table 3 depicts COVID-19 Impact indicators

## 4. Discussion

The present study aimed to evaluate the impact of the COVID-19 pandemic on children with ASD and ADHD in Northern Greece. Our findings offer a valuable contribution to the growing body of international literature, reflecting both consistencies and notable differences with previously reported trends.

A pronounced male predominance (77.8%) was observed in our sample, consistent with existing epidemiological evidence suggesting that ASD and ADHD are significantly more prevalent in males, with male-to-female ratios ranging from 3:1 to 4:1 [17,18]. The age distribution of our participants was centered around early and middle childhood (ages 5–9), aligning with global data indicating that this is the typical period for diagnosis and intervention initiation [19]. Although age is not a determining factor in the pathogenesis of neurodevelopmental disorders, awareness and formal diagnoses tend to increase in school-age children.

Contrary to studies suggesting that social or ethnic background may influence prevalence rates or access to diagnosis [20], our findings support a more uniform distribution: 94% of participants were of Greek origin, but diagnostic rates did not appear to vary significantly by ethnicity or geographic region. Similarly, no significant variation was found between urban, suburban, or rural settings, confirming findings from other studies suggesting that neurodevelopmental conditions transcend geographic and socioeconomic boundaries when access to healthcare is relatively consistent [21].

Regarding co-morbidities, learning difficulties (33.3%) were the most frequently reported, followed by anxiety disorders (8.3%) and epilepsy (6.9%). These results align with previous research indicating that children with ASD and ADHD frequently exhibit co-occurring conditions, particularly learning and emotional-behavioral disorders [22,23]. In our sample, approximately 60% of children had at least one co-occurring condition, broadly consistent with prior reports in autistic populations (e.g., Simonoff et al.), though estimates vary by ascertainment method and the range of conditions assessed [24]. The 40% reporting no additional diagnoses in our parent-reported data may reflect under-ascertainment, the narrower set of co-morbidities captured by our instrument, and the small, regional sample.

In terms of psychological functioning during the pandemic, 69.4% of parents described their child’s emotional state as “satisfactory,” while only 15.3% rated it as “poor.” These findings contrast with several studies that reported marked emotional and behavioral deterioration during the pandemic, particularly in children with ASD [12,13]. Notably, 43.1% of our participants reported new behavioral symptoms such as irritability, anxiety, and emotional dysregulation—figures lower than those reported in similar cohorts in Italy, Spain, or the UK, where such problems were observed in 60–75% of children [4,6,25]. This may reflect regional differences in family coping mechanisms, social support structures, or therapeutic continuity.

Caregiver well-being also deviated from international trends. While 29.2% of parents in our study experienced psychological distress, others report rates exceeding 50% among caregivers of neurodivergent children [26]. Only 40.3% of our sample sought psychological support during the pandemic, possibly due to perceived stigma or limited access to specialized services in certain areas—a barrier also identified in other European contexts [3].

Therapy disruptions emerged as a consistent concern, with nearly half of respondents (45.8%) reporting that their child’s therapy was reduced by more than 70%. This finding aligns with those of Jeste et al. (2020), who documented similar interruptions in autism services across North America and Europe [27]. Although 29.2% of families utilized online therapy, the majority expressed dissatisfaction with its effectiveness. These observations echo broader concerns in the literature regarding the limitations of telehealth for children with neurodevelopmental disorders, including reduced engagement, difficulty replicating structured behavioral interventions, and lack of trained parental facilitation [28,29].

A significant majority (87.5%) of parents perceived the pandemic as having negatively affected their child. This is comparable to international findings, although some studies also reported a minority of parents observing temporary improvements, such as reduced sensory overload or school-related stress [30]. The absence of such reports in our study may be related to the limited availability of structured remote learning or supportive home environments.

Screen time increases were nearly universal, with over 90% of parents reporting moderate to significant rises. This mirrors global trends documented during the pandemic, with increased device use linked to attentional difficulties, emotional dysregulation, and delayed sleep onset [31,32]. Surprisingly, only 22.2% of parents sought additional academic support for their children, in contrast to data from the U.S. and U.K., where remote tutoring and supplemental learning services became widespread [33]. This gap may reflect financial barriers or reduced parental availability due to work or caregiving demands.

Beyond the main trends discussed above, several additional findings merit further consideration. Sleep disturbances were reported in nearly one quarter of children, alongside the emergence of neurological or speech-related symptoms such as new-onset tics and stuttering in a minority of cases. These manifestations are consistent with previous evidence indicating that stress, disrupted routines, and increased screen exposure during the pandemic may exacerbate sleep dysregulation and neurodevelopmental vulnerabilities in children with ASD and ADHD [12,31,32]. Although these proportions are lower than some international reports [4,6], they highlight the need for targeted sleep hygiene and neurodevelopmental monitoring during periods of systemic disruption.

Parent–child interactions also emerged as a relevant domain: nearly half of families reported spending quality time with their children during lockdown, yet a considerable proportion indicated that this time was only moderately productive. Previous qualitative studies have suggested that while increased time at home may provide opportunities for closer bonding, it can also heighten stress in families with neurodiverse children, depending on support structures and caregiver resources [3,30]. Our findings support this nuanced picture, underscoring the importance of family-centered interventions.

Moreover, only a minority of parents accessed mental health services for their children, despite nearly one-third reporting significant psychological distress themselves. Limited uptake may reflect structural barriers, stigma, or service shortages—factors documented in other European settings [3,26]. Similarly, the low proportion of families seeking supplementary educational support contrasts with trends in other countries [33], potentially reflecting financial constraints or differences in remote education infrastructure.

Demographic characteristics such as parental education level and residential location did not appear to significantly influence service access or perceived impact, aligning with previous studies suggesting that when healthcare access is relatively consistent, neurodevelopmental challenges affect families across socio-economic and geographic boundaries [20,21].

Finally, economic resilience was variable: while 61.1% of families reported financial stability, 37.5% experienced economic decline—a figure consistent with studies noting disproportionate financial strain among families of children with disabilities during the pandemic [34]. Only one family (1.4%) reported financial improvement, underscoring the long-term socioeconomic consequences of the crisis.

The findings of this pilot study provide useful insights for refining the questionnaire design. In future iterations, clearer and more structured instructions for participants will be incorporated to enhance comprehension and data quality. Supplementary Materials are available online.

## 5. Limitations

This study has certain limitations that should be acknowledged. First, the relatively small sample size and its recruitment from therapeutic centers and Creative Activity Centers for Children in Northern Greece may limit the generalizability of the findings to broader populations of children with neurodevelopmental disorders. Given the pilot nature and modest sample size (N = 72), no a priori power calculations were performed. Post hoc estimations indicate that, assuming medium effect sizes (Cohen’s d = 0.5) and α = 0.05, the current sample would yield approximately 40–50% statistical power for two-group comparisons. A future study aiming for 80% power would require approximately 64 participants per group (N ≈ 128), which will guide the design of subsequent confirmatory analyses. Second, the cross-sectional design precludes causal inferences regarding the long-term effects of the COVID-19 pandemic. Third, the use of a structured questionnaire, although specifically designed and ethically approved, relied on parental reporting, which may be subject to recall bias and subjective interpretation. Finally, the study was confined to a six-month period between December 2022 and June 2023, and therefore cannot capture potential changes in the post-pandemic adjustment of children and their families over a longer timeframe. Despite these limitations, the study provides valuable insights into the enduring developmental, psychological, and social consequences of the pandemic in vulnerable pediatric populations.

## 6. Conclusions and Unique Contribution

In this post-pandemic, cross-sectional study of children with ASD and ADHD, families reported substantial therapy disruptions, low uptake and limited perceived effectiveness of online services, frequent screen-time increases, and elevated caregiver strain. Approximately 60% of children had ≥1 co-occurring condition, in line with the previous literature, underscoring the clinical complexity of care. These findings, aligned with our discussion, highlight the need to stabilize access to core therapies, strengthen caregiver supports, and standardize telehealth when in-person services are constrained. Given the design and sample size, results should be interpreted as associative rather than causal; nonetheless, they delineate actionable service gaps for health and education systems. Future research should adopt longitudinal designs, include validated outcome measures beyond parent report, and evaluate implementation strategies that sustain therapeutic continuity during system shocks.

By acknowledging the methodological and contextual constraints of this early research stage, this study contributes to the development of more robust future research designs in similar populations.

## Figures and Tables

**Table 1:** Demographic Characteristics.

Category	Subcategory
Gender	Boys: 56 (77.8%)/Girls: 16 (22.2%)
Age Group	2–5 yrs: 17 (23.6%)/5–9 yrs: 36 (50%)/9–13 yrs: 9 (12.5%)/13–17 yrs: 10 (13.9%)
Educational Status	Not attending: 9 (12.5%)/Primary: 53 (73.6%)/Secondary: 10 (13.9%)
Residence	City: 29 (40.3%)/Town: 28 (38.9%)/Village: 15 (20.8%)
Parental Education	Primary: 3 (4.2%)/Secondary: 12 (16.7%)/Tertiary: 57 (79.2%)

**Table 2:** Diagnostic and Comorbidity Profile.

Diagnosis/Comorbidity	Number (%)
ASD	57 (79.2%)
ADHD	15 (20.8%)
Learning difficulties	24 (33.3%)
Anxiety	6 (8.3%)
Epilepsy	5 (6.9%)
Multiple comorbidities	8 (11.1%)
No comorbidity	29 (40.3%)

**Table 3:** COVID-19 Impact Indicators.

Impact Area	Details
Child psychological state	Very good: 11/Satisfactory: 50/Poor: 11
Parent psychological state	Very good: 9/Satisfactory: 42/Poor: 21
Need for psychologist	Yes: 29 (40.3%)/No: 43 (59.7%)
Therapy reduction >70%	33 (45.8%)
New behavioral issues	Yes: 33 (45.8%)/No: 39 (54.2%)
Screen time increase	Marked: 34/Moderate: 32/No change: 6
Access to online therapy	Yes: 21 (29.2%)/No: 51 (70.8%)
Financial deterioration	27 (37.5%)

## Data Availability

All data supporting the findings of this study are deposited in the institutional repository of the University of Ioannina (https://lib.uoi.gr/ilektronikes-piges/idrumatiko-apothetirio-olumpias/). Any restrictions on data availability, such as those related to confidentiality, will be clearly indicated. Generative AI tools (OpenAI GPT-5) were used to assist with English-language editing and refinement of the manuscript text. No AI tools were employed in data collection, statistical analysis, or interpretation.

## Supplementary Materials

The following supporting information can be downloaded at: https://www.mdpi.com/article/10.3390/brainsci15111212/s1.

## Abbreviations

The following abbreviations are used in this manuscript:

ASD	Autism Spectrum Disorder
ADHD	Attention-Deficit/Hyperactivity Disorder
CACC	Creative Activity Centers for Children
