# Supplementary material for: Impact of the COVID-19 Pandemic on Children with ASD and ADHD in Northern Greece: A Pilot Study
Source: Brain Sci. 2025 Nov 10;15(11):1212. doi: 10.3390/brainsci15111212 (PMC12650278; doi:10.3390/brainsci15111212)
Supplement: Supplementary file 1 [file brainsci-15-01212-s001.zip › brainsci-3856120-supplementary.pdf]

Supplementary File — Questionnaire (English Translation)

Impact of the COVID-19 Pandemic on Autism and ADHD (Mid-pandemic Period)

Instructions: Please tick one option unless otherwise indicated. Where noted, multiple selections are allowed.

1. Please specify your child's sex:

- ☐Boy
- ☐Girl

2. Which age group does your child belong to?

- ☐2–5 years
- ☐5–9 years
- ☐9–13 years
- ☐13–17 years

3. Is your child of Greek origin?

- ☐Yes
- ☐No (Please specify: __________)

4. Is your child enrolled in and attending school during the current academic year?

- ☐Not attending school
- ☐Primary education
- ☐Secondary education

5. The area where your child resides is:

- ☐Large city
- ☐Small town/city
- ☐Village

6. What is the highest level of education you (the respondent) have completed?

- ☐Primary
- ☐Secondary
- ☐Tertiary

7. Which neurodevelopmental disorder has your child been diagnosed with?

- ☐Autism Spectrum Disorder (ASD)
- ☐AttentionDeficit/Hyperactivity Disorder (ADHD)

8. Does your child have any comorbid conditions? (select all that apply)

- ☐Epilepsy

- ☐Learning difficulties
- ☐Anxiety disorder

9. How would you rate your child’s overall psychological state during the COVID-19 pandemic?

- ☐Very good
- ☐Satisfactory
- ☐Not good at all

10. How would you rate your own overall psychological state during the COVID-19 pandemic?

- ☐Very good
- ☐Satisfactory
- ☐Not good at all

11. Did you feel the need to talk with a psychologist/psychiatrist about pandemic-related issues?

- ☐Yes
- ☐No

12. By what percentage do you believe your child’s therapeutic sessions decreased over the course of the pandemic (up to now)?

- ☐More than 70%
- ☐About 50%
- ☐Less than 30%

13. Were there any other behavioural problems that appeared for the first time during the pandemic period?

- ☐Yes
- ☐No

14. If yes, what type of behavioural problems were they? (select all that apply)

- ☐Irritability/aggressiveness
- ☐Anxiety
- ☐Emotional instability

15. Do you believe your daily routine was affected because of the pandemic?

- ☐Yes
- ☐No
- ☐Maybe

16. Did you undertake online/telehealth therapies because of the pandemic?

- ☐Yes

- ☐No

17. To what extent do you believe these (online/telehealth) therapies helped compared with in‑person sessions?

- ☐100%
- ☐Less than 50%
- ☐Not at all

18. During the pandemic, did your child's use of video gaming, mobile phone, or tablet increase?

- ☐Yes, to a great extent
- ☐Yes, but not much
- ☐Not particularly

19. Did you need to start additional private lessons to support your child at school?

- ☐Yes
- ☐No

20. During the pandemic, did you spend productive time together with your child?

- ☐Yes, to a great extent
- ☐Yes, but not as much as I expected
- ☐Not particularly

21. Do you believe that the conditions arising from the pandemic may have affected your child…

- ☐Negatively
- ☐Positively
- ☐Not at all

22. Did your child present sleep disturbances during the pandemic?

- ☐Yes
- ☐No

23. During the pandemic, did your child present for the first time any of the following? (select all that apply)

- ☐Tics
- ☐Stuttering
- ☐Regression of speech

24. Did you need to visit a child psychologist/child psychiatrist for your child due to pandemic-related circumstances?

- ☐Yes, but only once
- ☐Yes, on a regular basis

- ☐No

25. The family's financial situation during the pandemic:

- ☐Deteriorated
- ☐Remained stable
- ☐Improved

Note: This is an official English translation of the original Greek instrument used in the study.
